# Microglia Exhibit Distinct Heterogeneity Rather than M1/M2 Polarization within the Early Stage of Acute Ischemic Stroke

**DOI:** 10.14336/AD.2023.0505

**Published:** 2023-12-01

**Authors:** Hongyu Ma, He Li, Yongxin Zhang, Yu Zhou, Hanchen Liu, Hongye Xu, Luojiang Zhu, Guanghao Zhang, Jing Wang, Zifu Li, Bo Hong, Wang Zhou, Pengfei Yang, Jianmin Liu

**Affiliations:** ^1^Neurovascular Center, Changhai hospital, Naval Medical University, Shanghai, China, 100433; ^2^Emergency Department, Naval Hospital of Eastern Theater, Zhoushan, Zhejiang, China, 316000

**Keywords:** Microglia, acute ischemic stroke, cerebral ischemia, neuroprotection, single-cell RNA sequencing

## Abstract

The classification of microglial M1/M2 polarization in the acute phase of ischemic stroke remains controversial, which has limited further advances in neuroprotective strategy. To thoroughly assess the microglial phenotypes, we made the middle cerebral artery occlusion model in mice to simulate the acute pathological processes of ischemic stroke from normal conditions to acute cerebral ischemia and then to the early reperfusion period. The temporal changes in gene profiles, cell subtypes, and microglial function were comprehensively analyzed using single-cell RNA sequencing. We identified 37,614 microglial cells and divided them into eight distinct subpopulations. Mic_home, Mic_pre1, and Mic_pre2 subpopulations were three clusters mainly composed of cells from the control samples, in which Mic_home was a homeostatic subpopulation characterized by high expression of Hpgd and Tagap, and Mic_pre1 and Mic_pre2 were two clusters with preliminary inflammatory activation characteristics marked by P2ry13 and Wsb1 respectively. Mic_M1L1 and Mic_M1L2 subpopulations exhibited M1-like polarization manifested by the upregulation of inflammatory genes after ischemic stroke, while the intrinsic heterogeneity on the level of inflammatory responses and neurotrophic support properties was observed. Moreover, we identified three unique clusters of cells with low inflammation levels. Mic_np1, Mic_np2, and Mic_np3 were characterized by high expression of Arhgap45, Rgs10, and Pkm respectively. However, these cells did not show significant M2-like characteristics and their classic microglia function was also attenuated. These subpopulations exhibited higher activation of neuropeptide functional pathways. At last, we performed cell-cell communication analysis and identified major couplings contributing to the interaction between microglia and other cell populations. In summary, our study elucidated the temporal heterogeneity of microglia in the acute phase of ischemic stroke, which may facilitate the identification of effective neuroprotective targets to curb ischemic damage at an early stage.

## INTRODUCTION

Microglia are the resident immune cells of the central nervous system (CNS), constituting approximately 0.5% to 16.6% of all brain cells [1, 2]. As the first line of defense against central nervous system (CNS) injury, microglia play important roles in the acute stage of ischemic stroke [3]. After ischemic attack, microglia immediately activate and migrate to the lesion site [4]. They can promote CNS repair and regeneration, while also produce proinflammatory cytokines and cytotoxic substances that may exacerbate tissue injury [5, 6]. The dual functions of microglia, which exhibit both beneficial and detrimental effects, have led to the traditional binary classification of microglia by M1 or M2 phenotypes. With the increased discovery of new microglia functions, this hypothesis has been controversial for many years because it cannot comprehensively explain the complex physiology of microglia [7].

Recently, the use of single-cell RNA sequencing (scRNA-seq) has promoted the exploration of the regional and developmental heterogeneity of microglia [8-12]. For example, the discovery of the border-associated macrophage (BAM) and disease-associated microglia (DAM) has shed a new light on the diversity of microglia under specific disease conditions [13, 14]. Furthermore, previous studies have uncovered the phenotypes of microglia 24 hours after the reperfusion of ischemic stroke in mice [15-17]. However, the infarction region and neurological deficits have stabilized at 24 hours, which means that the ischemic penumbra might have been largely replaced by infarction cores, especially for severe stroke. According to clinical experience, the intervention in the first few hours before and after stroke has huge impact on long-term outcomes, which means that pathological process changes sharply during these periods. Moreover, damages after stroke, such as inflammatory injury, are usually characterized as cascade amplification; thus, knowing the early environment is beneficial to suppress the onset of inflammatory cascade. Since microglia are the first cells to respond to injury, further exploration of their immediate responses and phenotype states after cerebral ischemia and reperfusion is very important.

In this study, we performed scRNA-seq using brain cortices from mice that received sham surgery and mice who underwent two hours of middle cerebral artery occlusion, from which brain cortices were collected at three time points (no reperfusion, 2h after reperfusion, 12h after reperfusion). We aimed to elucidate the temporal heterogeneity of microglia and identify potential regulators, which may facilitate the exploration of useful neuroprotective strategies for alleviating the exacerbation of ischemic stroke at an early stage of the acute phase.

## MATERIALS AND METHODS

### Animals and group assignment

Our study has been approved by the ethics committee of the First Affiliated Hospital of Naval Medical University. All the animal operations and experimental procedures were carried out following the animal care standards of the National Institutes of Health Guide. Eight-week-old C57BL/6N male mice weighing 20-24g were acquired from the Beijing Vital River Company. The reared environment was a pathogen-free SPF animal room at 20-26 ° and 40-60% humidity, with a 12h light/dark cycle. Animals were randomly assigned into four groups named ‘control’, ‘pMCAO_2h’, ‘tMCAO_2h’, and ‘tMCAO_12h’ corresponding to the sham group and three intervention groups.

### Surgical procedures

Animals in the interventional groups received middle cerebral artery occlusion (MCAO) operation. In brief, anesthesia was induced using 5% isoflurane inhalation, followed by the continuous mask inhalation of 2.0% isoflurane for the maintenance of anesthesia. The operation was performed on a heat mat to keep the mice’s body temperature at 36.5 ± 0.5 °C. After shaving the hair on the neck and disinfection, we made a para-midline incision to expose the left common carotid artery (CCA), internal carotid artery (ICA), and external carotid artery (ECA). Then we cut a small incision on the ECA and insert a suture-embolus (Jialing Company, Guangzhou, China) into the ECA. We guided the suture-embolus through the carotid bifurcation to the ICA and pushed forward until a slight resistance was felt (approximately 9-10μm), in which the middle cerebral artery can be totally occluded. The change in the cerebral blood flow was monitored by the laser speckle flowmetry. After keeping focal cerebral ischemia (CBF<30%) for 2 hours, the suture-embolus was withdrawn for reperfusion. Mice in the control group merely received sham surgery, with the same anesthesia and surgical exposure but no suture-embolus insertion. In our experiment, the control state represented the healthy brain, while two hours of permanent MCAO samples (pMCAO_2h) can be regarded as a severe ischemic brain without blood reperfusion, two hours of transient MCAO followed by reperfusion for two hours simulated the ultra-early phase after reperfusion of ischemic stroke (tMCAO_2h), and transient MCAO with reperfusion for 12 hours represented the time point after a short period of blood reperfusion (tMCAO_2h).

### Single-cell isolation preparation

Mice were anesthetized using 5% isoflurane. Cold Phosphate Buffered Saline (PBS) was perfused through the left heart ventricle until the mice’s liver turned white. Brains cortices were extracted from mice. The brain tissues were triturated in Dulbecco's Phosphate Buffered Saline (DPBS, without calcium ions) and spun at 300g for 5 minutes. The supernatant was discarded, and the pellet was mixed with digestive enzymes. After adequate digestion, the suspension was filtered using 70μm cell strainers, then washed in DPBS again and centrifuged at 300g for 10 minutes. The erythrocytes were cleared away with lysis buffer. To remove the myelin sheath of the brain cells, the pellet was resuspended with 1 ml DPBS, and mixed with 2.1 ml DPBS and 900μl Debris Removal Solution. The mixture was covered with 4ml DPBS and centrifuged at 3000g for 10 minutes. Cell sediment in the lowest layer was kept and resuspended using DPBS and Bovine Serum Albumin Solution (BSA). A total of twelve samples were prepared for scRNA-seq, consisting of three biological replicates for each mouse group.

### 10x genomic single-cell RNA sequencing

Using 10x Genomics Platform, a single-cell library was constructed under the manufacturer’s instruction of Chromium Next GEM Single Cell V(D)J Reagent Kits v1.1.(10x Genomics, San Francisco, CA) Firstly, the single-cell gel beads-in-emulsion (GEM) was made by combining Master Mix with brain cell, barcoded Single Cell VDJ 5’ Gel Beads (v1.1), and partitioning oil using Chromium next GEM chip G. Then the GEM was dissolved and incubated to acquire 10x Barcoded full-length cDNA. Filtered with Silane magnetic beads, the cDNA was amplified to generate multiple libraries. To construct the 5' Gene Expression (GEX) library, the P5 primer, P7 primer, and Illumina R2 sequence (read 2 primer sequence) were added via end repair, A-tailing, adaptor ligation, and sample index PCR. The final libraries were sequenced using the Illumina Nova6000 platform with a sequencing depth of at least 100,000 reads per cell. The output of feature barcode matrices was generated using 10x Genomics Cell Ranger (v4.0.0) pipeline.

### Quality control and clustering

Analysis of scRNA-seq data was performed on R (v4.0.3) platform. Quality control of raw data from each sample was processed primarily with the ‘Seurat’ package (v4.1.1, https://satijalab.org/seurat). Low-quality genes or cells were filtered using the following standards: 1. The genes were expressed in no more than 3 cells; 2. The cells contained more than 20% mitochondrial transcripts or less than 5% ribosomal transcripts; 3. The cells expressed less than 200 genes, or more than 5000 genes, or doublets identified by ‘DoubletFinder’ packages [18]. The filtered data were normalized using ‘NormalizeData’ function. The 1500 variable genes screened by ‘FindVariable Features’ function were utilized in the principal component analysis. After quality control procedures, the Seurat datasets from twelve samples were integrated via ‘RunHarmony’ function. A shared nearest neighbor graph (SNN) was constructed by ‘FindNeighbors’ function. The dimension reduction was performed using ‘harmony’ function with a 30 dimensions input [19]. Uniform Manifold Approximation and Projection (UMAP) were plotted to display the unsupervised clustering results. The brain cell populations were identified and manually labeled based on the expression of marker genes reported by the literature. Finally, the dimensionality reduction and re-clustering were performed using ‘harmony’ method (v1.0) together with ‘monocle3’ package (v0.2.3.0) to further divide the subpopulations of microglia [20].

### Percentage of cells per cluster/sample analysis

Subpopulations were annotated for microglia pathology status and phenotypic traits by assessing the overexpressed genes and the corresponding functional traits. Proportional abundance at each time point was calculated for all subpopulations. The cellular activation trajectories were constructed via pseudotime analysis, calculated by the DDR algorithm implemented in the ‘learn_graph’ function from monocle3 package. Pseudotime ordering was performed by rooting the trajectory in the graph node with maximized distance among the activated cells. The top 10 high variable genes alongside the pseudotime trajectories were selected using the spatial correlation analysis and Moran’s I approach implemented in the ‘graph_test’ function.

### Differentially expressed genes and functional pathway analysis

Differentially expressed genes in each subpopulation were selected using the ‘FindAllMarker’ function with a ‘logfc’ threshold of 0.1, which represented the statistical significance of the expression of a gene in one subpopulation compared with the others, and minimal ‘pct’ value of 0.1, which means the percentages of cells that express a specific gene in one subpopulation. The values of the expression of specific genes in all subpopulations were displayed by dot plots and violin plots. Differentially expressed genes between two selected clusters were compared using volcano plots. To assess the pathway activation in each subpopulation, gene set variation analysis (GSVA) was performed using gene sets of C2 and C5 collection obtained from the molecular signature database. Highly activated pathways related to microglia were visualized through heatmap plots. Variable expressed pathways were selected and compared within correlative subpopulations via violin plot. The connection among activated pathways in each subpopulation was analyzed using gene ontology network.

### Transcription factor regulatory network analysis

The transcription factor was analyzed using pySCENIC (python implementation of the Single-Cell Regulatory Network Inference and Clustering pipeline) [21]. The gene regulatory networks (GRN) were computed using the ‘grnboost2’ method. The enriched motif was identified with CisTarget databases (https://resources.aertslab.org/cistarget) containing the ‘mm9-tss-centered-10kb-7species.mc9nr.feather’ dataset and the transcription factor motif annotation database(v9). The regulon activities of all cells were scored by the ‘AUCell’ function. The similarity score of the regulons in each cluster was calculated and transferred to a specific score using Jensen-Shannon divergence. Heatmap plot was depicted to show the transcription factors with high activity in each subpopulation. Barplots were used to display the transcription factors with significant activation changes among the selected subpopulations.

### Cell communication analysis

Using the normalized count data derived from the Seurat subject, we performed ‘CellphoneDB’ algorithm (v3.0.1, https://www.cellphonedb.org) to analyze the cross-talk between subpopulations of microglia and other brain cell populations. Ligand-receptor pairs with a significant differential expression were selected and depicted in dot plots. The major interaction-couples and their functions were displayed with a schematic diagram. The principle and mechanism of the selection of ligands and receptors refer to the study by Garcia-Alonso et al [22].

### Pathological evaluation of ischemic regions

Coronal brain sections (2mm-thick) were acquired from mice at each designed time point. We performed triphenyltetrazolium chloride (TTC) staining to determine the infarcted volume of the mice after MCAO surgery. The brain sections were incubated in 2% TTC solution at 37° for 5min. The vital tissues would turn red, and the infarcted regions remain white. Brain sections were deposited in 4% paraformaldehyde for 24 hours and embedded in paraffin for sample store. The thickness of the paraffin slices was 4μm. To evaluate the ischemic injury to neural cells, Hematoxylin and eosin (HE) staining and Nissl staining were performed following the experimental kits from Biossci Technology Co. Ltd. (Wuhan, China) and the manufacturer's instructions.

### Immunofluorescence

Double immunofluorescent staining was performed to verify the subpopulations of microglia. The brain sections were deparaffinized using xylene and rehydrated with gradient ethanol (100%, 95%, and 75%). Swamping the section with boiled antigen repair solution (PH9.0; Ethylene Diamine Tetraacetic Acid) and heating for 90 seconds in a high-pressure boiler. After cooling down to room temperature, we put the sections into 3% H_2_O_2_ for 30min and washed them with distilled water. Then the sections were submerged in Tris-buffered saline (TBS) plus tween-20 solution (TBST) and blocked with 10% goat serum for 30min. Abandoning the serum, each brain section was covered with 50μl diluted Iba1 antibody solution (a marker of microglia; Abcam; ab178846; 1:10000) and incubated overnight. For double immunofluorescent staining, the incubated sections were washed with TBST solution, treated with 50μL Goat Anti-Rabbit IgG (HRP) antibody (Abcam; ab205718; 1:4000) solution, and incubated in 37° for 45min in the second day. The sections were then incubated with 50μL fluorescein Isothiocyanate (FITC; thermofisher; B40953; 1:300) for 10min and treated with antigen repair solution (PH6.0; Citrate Antigen Retrieval Solution). After the process of serum blocking, the antibody of the signature marker of each subpopulation was used for double staining, including Hpgd (the marker for Mic_home; Affinity; DF14850; 1:100), P2ry13 (the marker for Mic_pre1; allomone; APR-017; 1:100), Wsb1 (the marker for Mic_pre2; Affinity; DF12505; 1:100), Nfkbiz (the marker for Mic_M1L1; Affinity; DF12429; 1:100), Cd83 (the marker for Mic_M1L2; Affinity; AF5233; 1:400), Arhgap45(the marker for Mic_np1; proteintech; 14832-1-AP; 1:100), Rgs10 (the marker for Mic_np2;Affinity; DF4414; 1:100), and Pkm (the marker for Mic_np3; Affinity; DF5234; 1:100). The fluorescent antibody for these markers were Alexa Fluor®594 donkey anti-rabbit lgG(H+L) (life technologies; A21207; 1:400). The cell nuclei were stained with DAPI (Solarbio; C0060; 1:400). Finally, the stained cells were examined and analyzed using an inverted confocal microscope. For each cell marker, three biological duplicates of double-immunofluorescence staining were performed for each brain section (N=3). Two different regions (500μm^2^) surrounding the infarction core from each biological duplicate were selected for cell ratio counting (N=6). On the fluorescence figure, tissues without staining were black, live cell nuclei stained by DAPI were blue dots, microglia stained by Iba1 were green dots with surrounding tentacles, the marker proteins that were stained by corresponding antibodies gave off red light, and an isometric yellow bar at the bottom of the figure represented 100μm. Preliminary experiments were conducted to find the optimal concentration of antibodies. A secondary antibody only control was performed to avoid the nonspecifically bind to certain cellular compartments.

### Statistical analysis

The statistical methods were based on the R (v4.0.3) platform. Quantitative data were expressed as mean and standard error. Data of gene expression were normalized by the ‘NormalizeData’ function. Differential expression of genes among subpopulations was compared using Wilcoxon rank sum test implanted in the ‘Seurat’ package. The data of GSVA-score were assumed to be normal and were assessed using two-tailed unpaired student’s t-test. Statistical differences in cell counting were assessed using the nonparametric Wilcoxon test because the sample number in each timepoint was small. The number of biological and technical replicates is reported in figure legends. The P values were adjusted by Bonferroni Correction.

**Figure 1.**
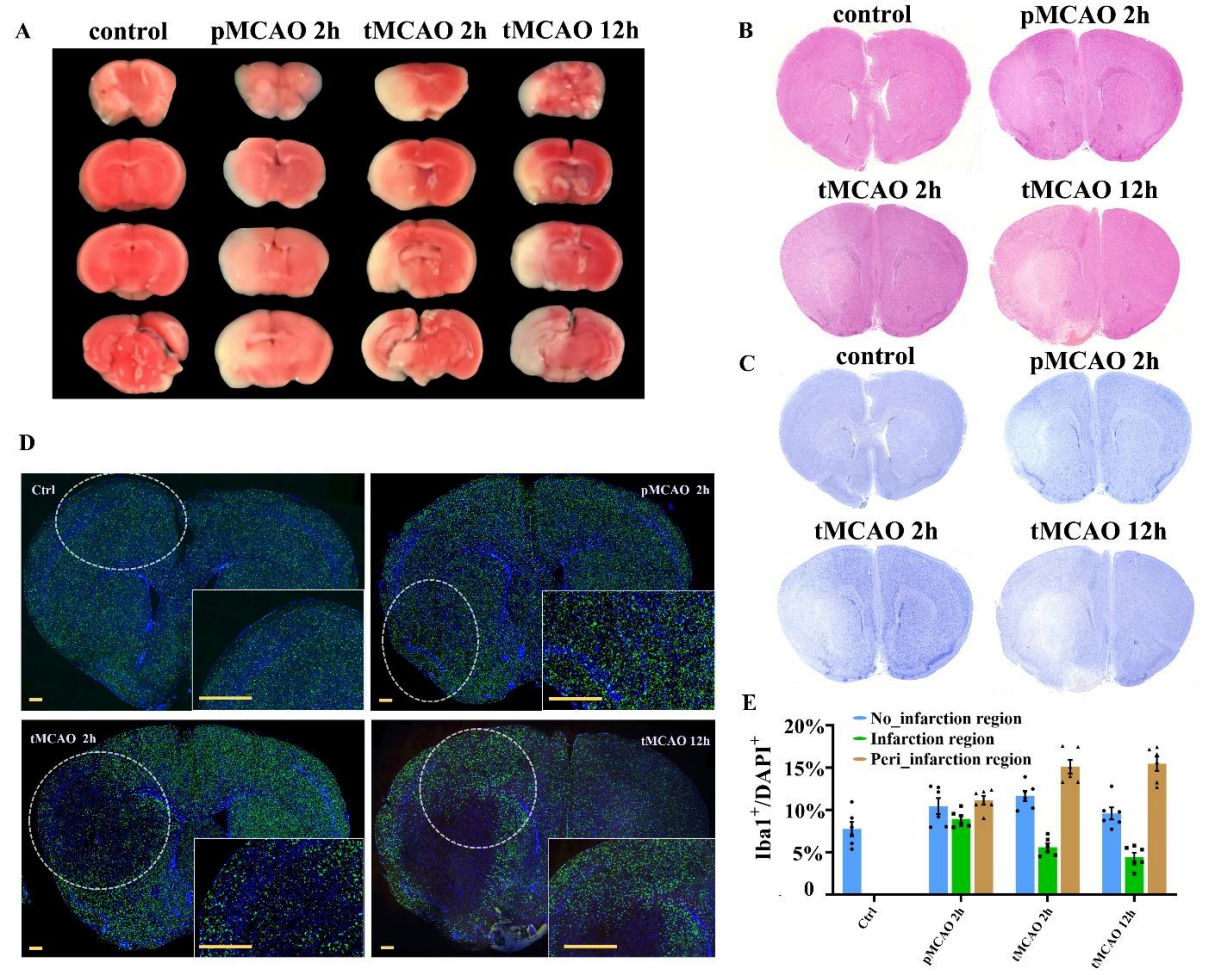
Pathological changes of brain cortices sections after cerebral ischemia and reperfusion. (A) Triphenyltetrazolium chloride (TTC) staining of the infarcted (white) and vital (red) tissues in the brain sections at four different sample time points. (B) Hematoxylin and eosin (HE) staining shows changes in the pathological structures of the brain sections. N=3. (C) Nissl staining reveals the vitality of neurons. N=3. (D) Immunofluorescence staining using Iba1 and DAPI antibodies displays the temporal dynamics in the distribution of microglia. N=3. The yellow bars represent 500μm. (E) The ratio of Iba1^+^ DAPI^+^ microglia (green plus blue) to DAPI^+^ cells in a 500μm^2^ cortical area surrounding the infarction core (obtained from similar area in the sections from four time point). N=6.

## RESULTS

### The changes in pathological characteristics and microglia distribution during the very early stage after reperfusion

The temporal change of environment at the early stage of acute ischemic stroke was simulated by animal models. We first examined the changes in blood flow, infarcted regions, and histological characteristics of brain cortices from sample groups representing four early time points. The blood flow of the ischemic hemisphere ameliorated after the reperfusion but did not totally restore to the normal level before occlusion (Supplementary Fig. 1A). Similar to the study by Liu et al, the regions containing vital tissues stained by TTC solution was reduced with the ischemia and reperfusion progression [23] (Fig. 1A). HE staining and Nissl staining further verified the extension of the injured tissue structure and the reduction of neuron density (Fig. 1B, Fig. 1C). Notably, the distribution of microglia changed drastically (Fig. 1D). Before the reperfusion of ischemic stroke, the density of microglia showed no obvious change but was rapidly reduced in the infarcted region after blood reperfusion. Along with the reperfusion, fluorescence signals surrounding the infarction core were increasingly elevated, suggesting that microglia might have migrated and infiltrated the ischemic penumbra regions (Fig. 1E).

**Figure 2.**
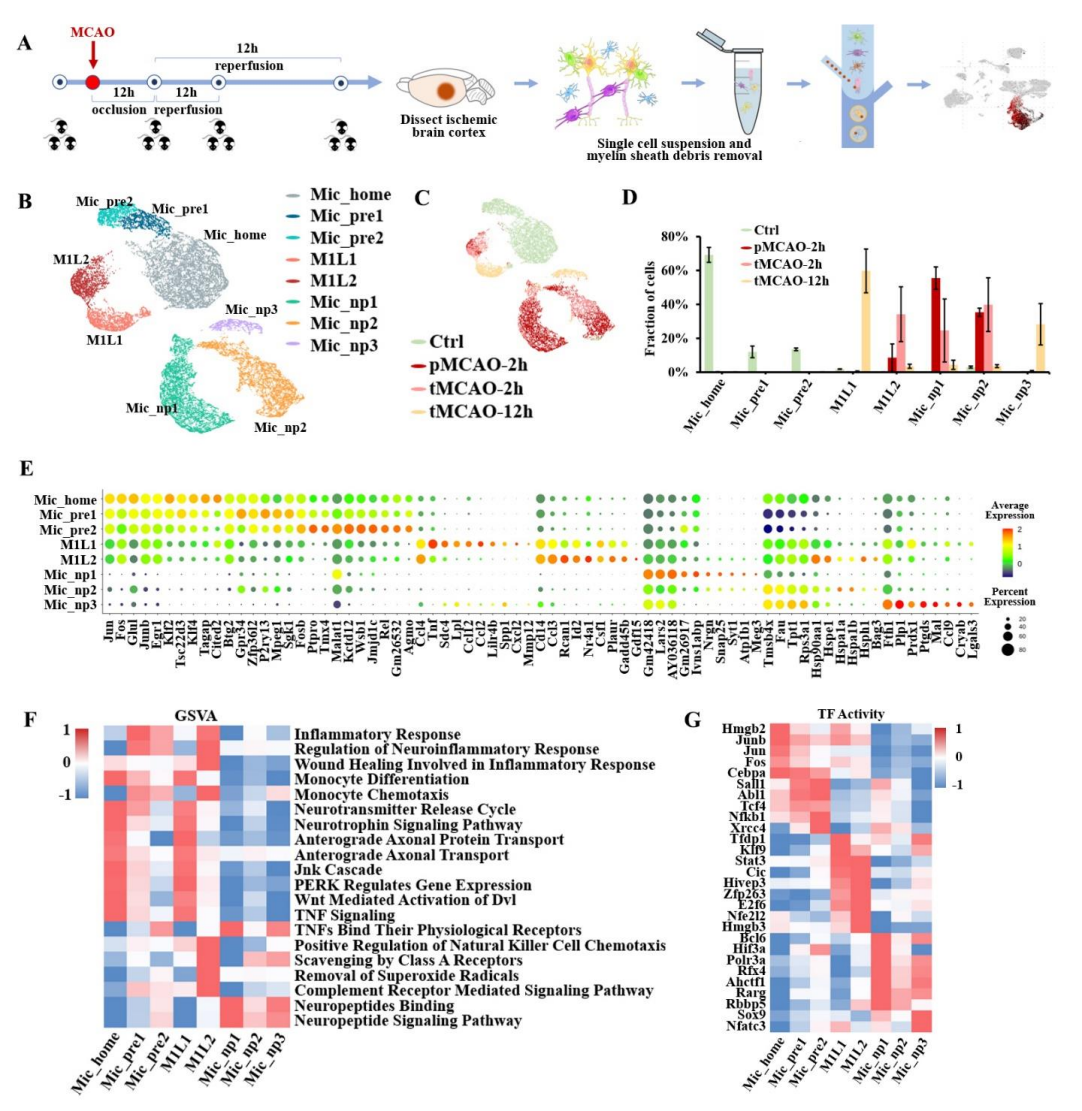
Transcriptional landscape of microglia at the acute stage of ischemic stroke. (A) Schematic diagram of sample acquisition, single-cell RNA sequencing, and microglia identification. (Staining by Tmem119). (B) Uniform Manifold Approximation and Projection (UMAP) plot of the microglial subpopulations. (C) The temporal distribution of microglial cells in the UMAP plot. (D) Bar plot of the temporal abundance proportion of each subpopulation. (E) Dot plot displays signature markers of each subpopulation. (F) Heatmap of gene set variation analysis (GSVA) pathway enrichment for each subpopulation. (G) Heatmap of highly activated transcription factors (TF) for each microglial subpopulation.

**Figure 3.**
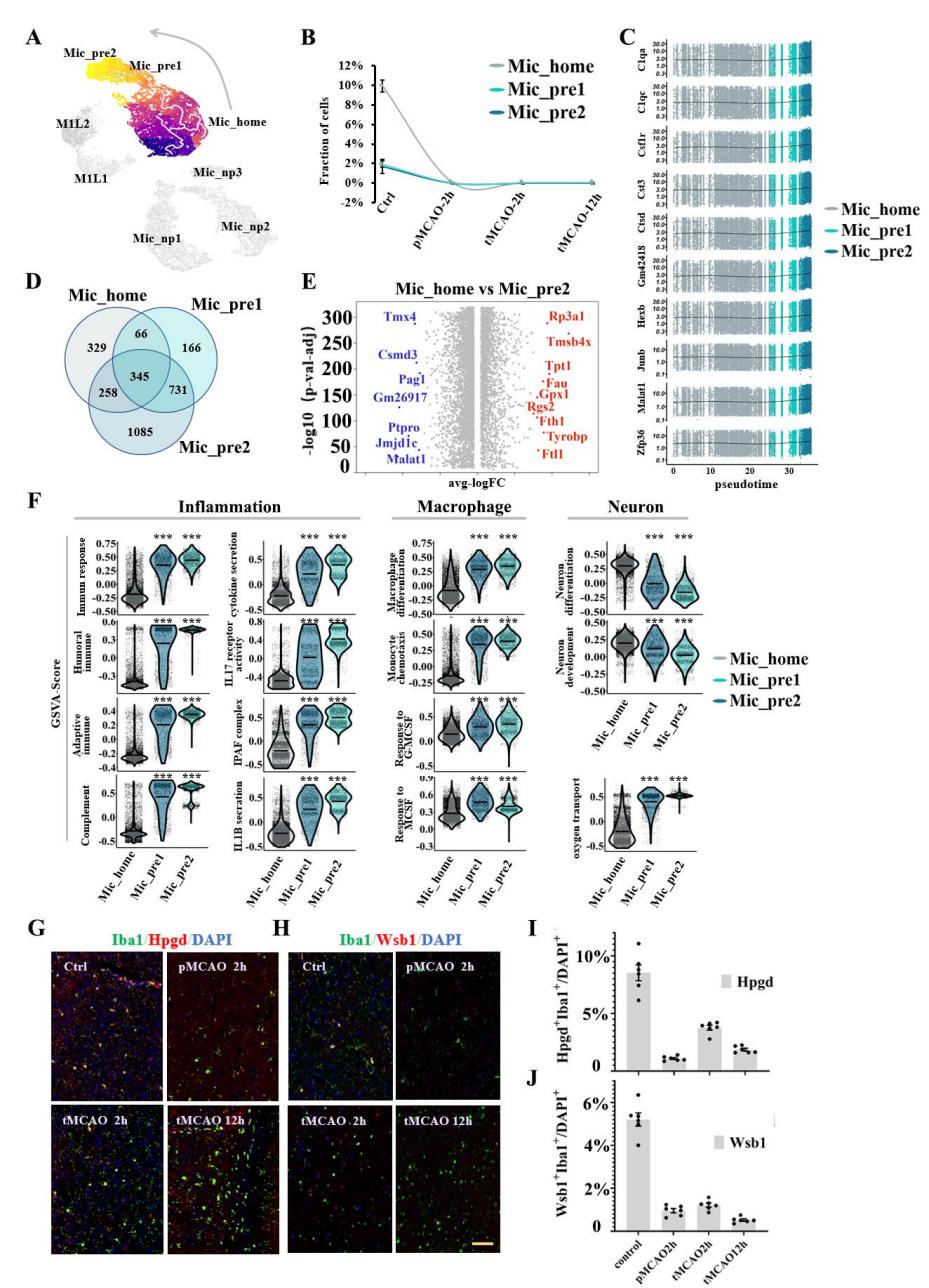
Subpopulation analysis of microglia clusters under homeostatic conditions. (A) Pseudotime plot (from monocle3) shows the differentiation trajectory from Mic_home to Mic_pre2 cells. (B) Line plot of the fraction of cells in the Mic_home, Mic_pre1, and Mic_pre2 subpopulations over time. (C) Expression of the ten genes with the highest Moran’s I score alongside the pseudotime trajectory. (D) Venn plot of the mutual and unique marker genes for the Mic_home, Mic_pre1, and Mic_pre2 subpopulations. (E) Volcano plot displays the differentially expressed genes between Mic_home and Mic_pre2 cells. Red dots represent the upregulated genes in the Mic_home cluster compared with the Mic_pre2 cluster and blue dots represent the downregulated genes. (F) Violin plot of the gene set variation analysis (GSVA) scores for important functional pathways of the Mic_home, Mic_pre1, and Mic_pre2 subpopulations. (G) Double immunofluorescence staining of Mic_home cells at each sampling time. Coronal brain sections are all stained with anti-Iba1(green, representing microglia), DAPI (blue, representing cell nuclei), and Hpgd (red, representing the marker of Mic_home cells) antibodies (N=3). The yellow bar represents 100μm. (H) Double immunofluorescence staining of Mic_pre2 cells at each sampling time. Coronal brain sections are all stained with anti-Iba1(green, representing microglia), DAPI (blue, representing cell nuclei), and Wsb1 (red, representing the marker of Mic_pre2 cells) antibodies (N=3). The yellow bar represents 100μm. (I) Bar plot of the ratio of Hpgd^+^Iba1^+^ cells (green plus red) compared with all DAPI^+^ cells in a 500μm^2^ area surrounding the infarction core (N=6). (J) Bar plot of the ratio of Wsb1^+^Iba1^+^ cells (green plus red) compared with DAPI^+^ cells in a 500μm^2^ area surrounding the infarction core (N=6).

### Microglia exhibit abundant phenotypes rather than binary polarization toward M1 and M2

According to the well-known hypothesis, microglia polarize toward M1 or M2 phenotypes. However, whether M1/M2 polarization is robust after ischemic stroke still needs further investigation [24]. Here, we used scRNA-seq to uncover microglial heterogeneity during this period. Cortical brain cells were isolated from mice that underwent MCAO or sham surgery. A total of 128,477 cells were collected after quality filtration. Cells were integrated using the ‘harmony’ package in R and visualized with the Uniform Manifold Approximation and Projection (UMAP). Brain cell populations were identified according to published cell markers, (http://bio-bigdata.hrbmu.edu.cn/CellMarker) in which microglia were labeled by “Tmem119”, “Cx3cr1”, “P2ry12”, and “Siglech” (Fig. 2A). In total, 37,614 cells were identified as microglia. Here, we revealed eight distinct subpopulations after statistical clustering using the ‘harmony’ and ‘monocle3’ packages (Fig. 2B). The proportional abundance of each subpopulation varied at the four time points (Fig. 2C, Fig. 2D). A dot plot was depicted to show the scaled expression of the signature genes of each cluster (Fig. 2E). We first examined the expression of classic markers for microglial polarization. According to the literature, there are three groups of markers corresponding to M1, M2a, and M2b/c phenotypes, whereas only a few of these markers were highly expressed in our system [24]. The representative genes for microglial polarization were displayed in Supplementary Fig. 1B and Supplementary Fig. 1C. Moreover, the marker genes from different polarization groups were coexpressed in one subpopulation, which may not support the simplistic binary classification of M1 and M2. This finding has also been reported in a traumatic brain injury study by Kim et al [25].

For the cell cycle changes, the G2/M phase increased after cerebral ischemia and decreased to a normal level 12 hours after reperfusion, implying the upregulation of cell proliferation genes induced by ischemic stroke. (Supplementary Fig.1D) Thereafter, we analyzed the subpopulations according to their signature genes, and each subpopulation was annotated according to its biological function. The activated functional pathways in each subpopulation were shown in a heatmap of GSVA enrichment (Fig. 2F). For further exploration, we calculated the scaled expression of genes for inflammation activation, such as chemokine cytokines and interleukin, and for anti-inflammation and neurotropic functions such as neurotrophic factor, growth factors, and neuropeptide. (Searching from ‘www.proteinatlas.org’ and ‘www.genecards.org’) (Supplementary Fig. 2). In addition, we performed single-cell regulatory network inference and clustering (SCENIC) analysis to identify the transcription factors that regulate the gene expression of each subpopulation. The most activated transcription factors in each subpopulation were presented in Fig. 2G. According to the results displayed in the heatmap, the above subpopulations could be categorized into four major groups: Mic_home (homeostatic microglia), Mic_pre (preliminary active microglia), Mic_M1L (M1-like-polarization microglia), and Mic_np (neuropeptide-secretion microglia). The expression of signature transcription factors was similar in each major cell population group. Furthermore, each cell category was dominated at its specific temporal interval (Supplementary Fig. 1E).

### Homeostatic and preliminary inflammation states exist before ischemic stroke

Rather than being transcriptionally homogeneous under normal conditions, three distinct subpopulations mostly consisting of cells from sham surgery samples were identified, which were Mic_home (homeostatic microglia), Mic_pre1 (preliminary active microglia type1), and Mic_pre2 (preliminary active microglia type2) (Fig. 3A, Fig. 3B). The subpopulation possessing the largest proportion of captured cells named Mic_home (n= 11259 cells, 29.9%). This subpopulation was characterized by a low inflammation level, abundant neurotrophic function, and high expression of Hpgd, Tagap, Crybb1, and Cited2. Additionally, GSVA enrichment revealed high monocyte differentiation signals and less monocyte chemotaxis in this subpopulation (Fig. 2F). At the same time, two other subpopulations in the system exhibited higher inflammatory levels. These cells showed a polarized tendency toward the M2 phenotype because of the moderate upregulation of Tgfb/Tgfbr and Mrc1 (Supplementary Fig. 1B). However, they had not been exposed to ischemic attack. Other classic markers for M2 polarization such as Il4, Il10, Il13, Cd163, and Arg1 were almost no expression. Hence, we named the two clusters Mic_pre1 (n= 1946 cells, 5.2%) and Mic_pre2 (n= 2208 cells, 5.9%), representing the preliminary active state. The signature genes of Mic_pre1 were Btg2, Gpr34, P2ry13, and Mpeg1, and those of Mic_pre2 were Ptpro, Kctd12, Wsb1, Jmjd1c, and Rel.

The pseudotime technique (from monocle3 package) revealed the differentiation trajectory of cells from Mic_home cluster to Mic_pre2 cluster (Fig. 3A). We used the ‘graph_test’ function (from monocle3 package) to select 10 genes with the highest Moran’s I score. The average expression of the 10 genes was stable and slightly upregulated at the end of the pseudotime trajectory (Fig. 3C). Moreover, the percentage of cells in S plus G2/M phases was approximately 30% lower in the Mic_pre clusters compared with the Mic_home cluster (Fig. 5C). The number of unique and shared signature genes among the three subpopulations was presented in the Venn diagram. Mic_pre2 cluster had the most unique genes (Fig. 3D). The volcano plot showed that genes related to protein synthesis (such as Tmx4) and immune cell regulation (such as Pag1 and Ptpro) were significantly upregulated in Mic_pre2 cells compared with Mic_home cells (Fig. 3E). The disparities in signature genes between Mic_home and Mic_pre1 cells as well as those between Mic_pre1 and Mic_pre2 cells were also displayed (Supplementary Fig. 3A, Supplementary Fig. 3B). We used violin plots to illustrate the differences in major functional pathways among the three subpopulations. Compared with the Mic_home cluster, the pathways for immune response, complement activation, and inflammatory cytokine secretion were significantly upregulated in Mic_pre1 and Mic_pre2 cells, while the expression for monocyte chemotaxis and the response to macrophage colony-stimulating factor (MCSF) were also evidently upregulated (Fig. 3F). In contrast, Mic_home cells were more associated with neural differentiation and development pathways. The ontology networks further illustrated the intrinsic interaction of functional pathways in each subpopulation. In addition to immune properties, Mic_home cells were found to have higher activation of neural secretion and glucose receptor production, while Mic_pre1 cells were of DNA damage recovery and aerobic respiration, and Mic_pre2 cells were associated with more intracellular signaling pathway activation and neurodegenerative signals (Supplementary Fig. 3C, Supplementary Fig. 3D, and Supplementary Fig. 3E). To further evaluate the transcriptional regulation of phenotype change, we selected seven transcription factors among the three subpopulations according to the extent of activity changes. (Supplementary Fig. 3F). The upregulation of Nfkb1, Bach2, Lef1, Gm14325, and Xrcc4, as well as the downregulation of Hmgb2, might induce microglia to be involved in inflammatory responses and DNA damage recovery. Double immunohistochemical staining was conducted to verify the biological changes in the gene profile of the cells (Fig. 3G, Fig. 3H). A dot plot of the expression of marker genes is shown in Supplementary Fig. 4A. The trends in the fraction of the signature genes of each subpopulation were in line with the results of scRNA-seq (Fig. 3I, Fig. 3J).

### Inflammatory-activated microglial subpopulations exhibit different biological phenotypes

**Figure 4.**
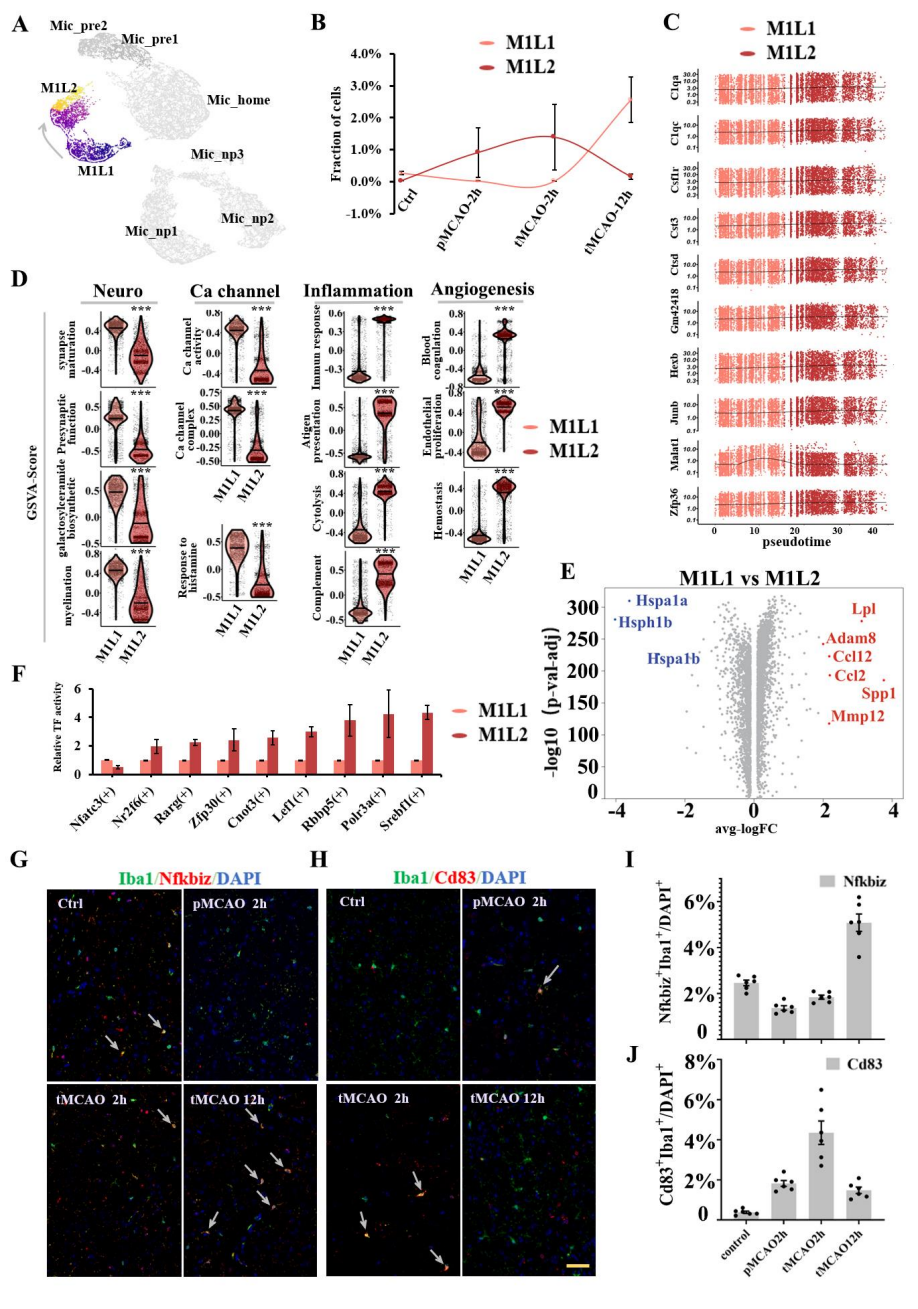
Subpopulation analysis of M1-polarization-like microglia clusters. (A) Pseudotime plot (from monocle3) shows the differentiation trajectory between Mic_M1L1 and Mic_M1L2 cells. (B) Line plot of the fraction of cells in Mic_M1L1 and Mic_M1L2 subpopulations over time. (C) Expression of the ten genes with the highest Moran’s I score alongside the pseudotime trajectory. (D) Violin plot of the functional pathways revealed by gene set variation analysis (GSVA) in the Mic_M1L1 and Mic_M1L2 subpopulations. (E) Volcano plot displays the differentially expressed genes between Mic_M1L1 and Mic_M1L2 cells. Red dots represent the upregulated genes in the Mic_M1L1 cluster compared with the Mic_M1L2 cluster and blue dots represent the downregulated. (F) Relative activity of the highly expressed transcription factors (TF) in Mic_M1L2 cells compared with Mic_M1L1 cells. (G) Double immunofluorescence staining of Mic_M1L1 cells at each sampling time. Coronal brain sections are all stained with anti-Iba1(green, representing microglia), Nfkbiz (red, the marker of Mic_M1L1 cells), and DAPI (blue, representing cell nuclei) antibodies (N=3). The yellow bar represents 100μm. The arrows point to the cells simultaneously marked by Iba1 and Nfkbiz. (H) Double immunofluorescence staining of Mic_M1L2 cells at each sampling time. Coronal brain sections are all stained with anti-Iba1(green, representing microglia), Cd83 (red, the marker of Mic_M1L2 cells), and DAPI (blue, representing cell nuclei) antibodies (N=3). The yellow bar represents 100μm. The arrows point to the cells simultaneously marked by Iba1 and Cd83. (I) Bar plot of the ratio of Nfkbiz^+^Iba1^+^ cells (green plus red) compared with all DAPI^+^ cells in a 500μm^2^ area surrounding the infarction core (N=6). (J) Bar plot of the ratio of Cd83^+^Iba1^+^ cells (green plus red) compared with all DAPI^+^ cells in a 500μm^2^ area surrounding the infarction core (N=6).

**Figure 5.**
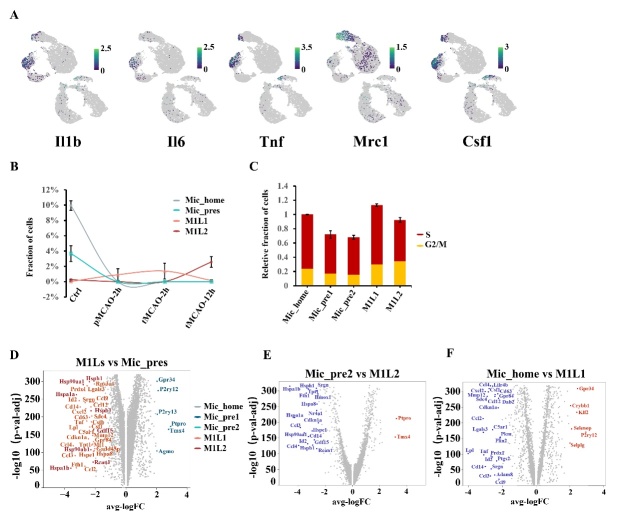
The comparison between M1-polarization-like subpopulations and clusters under homeostatic state. (A) Feature plot of the representative genes for M1 and M2 polarization. (B) Line plot to compare the proportional amount of Mic_home, Mic_pres, Mic_M1L1, and Mic_M1L2 cells over time. (C) Relative fraction of cells at S and G2/M phases in Mic_pre1, Mic_pre2, Mic_M1L1, and Mic_M1L2 subpopulations. The total value of the Mic_home is regarded as 1. (D) Volcano plot displaying the differentially expressed genes between Mic_M1Ls and Mic_pres clusters. The genes are shown in different colors according to the subpopulation with the higher expression. (E) Volcano plot displaying the differentially expressed genes between Mic_pre2 and Mic_M1L2 clusters. Red dots represent the upregulated genes in the Mic_pre2 cluster compared with the Mic_M1L2 cluster and blue dots represent the downregulated. (F) Volcano plot displaying the differentially expressed genes between Mic_home and Mic_M1L1 subpopulations. Red dots represent the upregulated genes in the Mic_home cluster compared with the Mic_M1L1 cluster and blue dots represent the downregulated.

Two subpopulations exhibiting significant activation of inflammatory genes were mainly identified in poststroke samples. From the perspective of microglial polarization, both clusters might be traditionally categorized as M1-polarization-like phenotypes due to the high expression of proinflammation genes such as Tnf, Lpl, Il1b, Csf1, and chemokines factors genes (Fig. 2E, Supplementary Fig.1B). Here, we uniquely named them Mic_M1L1 (M1-like-polarization microglia type1, n= 3245 cells, 8.6%) and Mic_M1L2 (M1-like-polarization microglia type2, n= 2801 cells, 7.4%) The fraction of Mic_M1L2 cells continued to increase within 2 hours after reperfusion but decreased in a few hours later. On the other hand, the fraction of Mic_M1L1 cells was dominated at 12 hours after reperfusion (Fig. 4A, Fig. 4B). Mic_M1L1 cells were characterized by marker genes including Lpl, Mydgf, Nfkbiz, Ccl2, and Spp1, while Mic_M1L2 cells were characterized by genes including Cd83, Rcan1, Nr4a1, Ccl3, and Id2. The intrinsic differentiation connection between Mic_M1L1 and Mic_M1L2 was predicted by monocle3 algorithm. The trajectory development was associated with the intensity of cells with high expression of Il1b, Il6, Tnf, and Csf1 (Fig. 4A, Fig. 5A). The genes with high Moran’s I score in the two clusters were continuous, suggesting that the differentiation procedure might be consecutive (Fig. 4C).

The average expression level of inflammatory factors such as Tnf, Lpl, Ccl2, Ccl12, and Cxcl2 was higher in the Mic_M1L1 cluster (Fig. 2E, Supplementary Fig.1B), but a large portion of the cells with the highest expression of the above genes pertained to the Mic_M1L2 cluster (Fig. 5A). Besides inflammatory pathways, Mic_M1L1 cells were also associated with elevated calcium channel activity and neural support function, including synapse maturation, presynaptic function, galactosylceramide biosynthesis, and myelination; conversely, their inflammatory function was downregulated compared with Mic_M1L2 cells (Fig. 4D). In addition, the hemostasis and blood coagulation pathways were also upregulated in Mic_M1L2 cells (Fig. 4D). Differentially expressed genes between Mic_M1L1 and Mic_M1L2 were compared using the volcano plot (Fig. 4E).

The above changes in the gene profiles between Mic_M1L1 and Mic_M1L2 cells might be influenced by the activity of transcription factors including Srebf1, Polr3a, Rbbp5, Lef1, Cnot3, Zfp30, Rarg, Nr2f6, and Nfatc3 (Fig. 4F). These transcription factors were significantly upregulated in Mic_M1L2 cells and promoted functions associated with lipid metabolism, innate immune response, and cell proliferation. Double immunohistochemical staining further verified the trends in the abundance of signature markers of Mic_M1L1 and Mic_M1L2 cells (Fig. 4G, Fig. 4H, Fig. 4I, and Fig. 4J). The temporal change of the fraction of Mic_M1L cells were very different from the Mic_home and Mic_pre cells (Fig. 5B). Compared with the Mic_home and Mic_pre clusters, the fraction of cells in S and G2/M phases in Mic_M1L clusters were increased (Fig. 5C). Volcano plots analysis suggested that the highly expressed differential genes in Mic_M1L cells were not similar to the preliminary activated clusters (Fig. 5D, Fig. 5E, and Fig. 5F).

### Neuropeptide-associated microglia with distinct characteristics were uncovered during acute ischemic stroke

**Figure 6.**
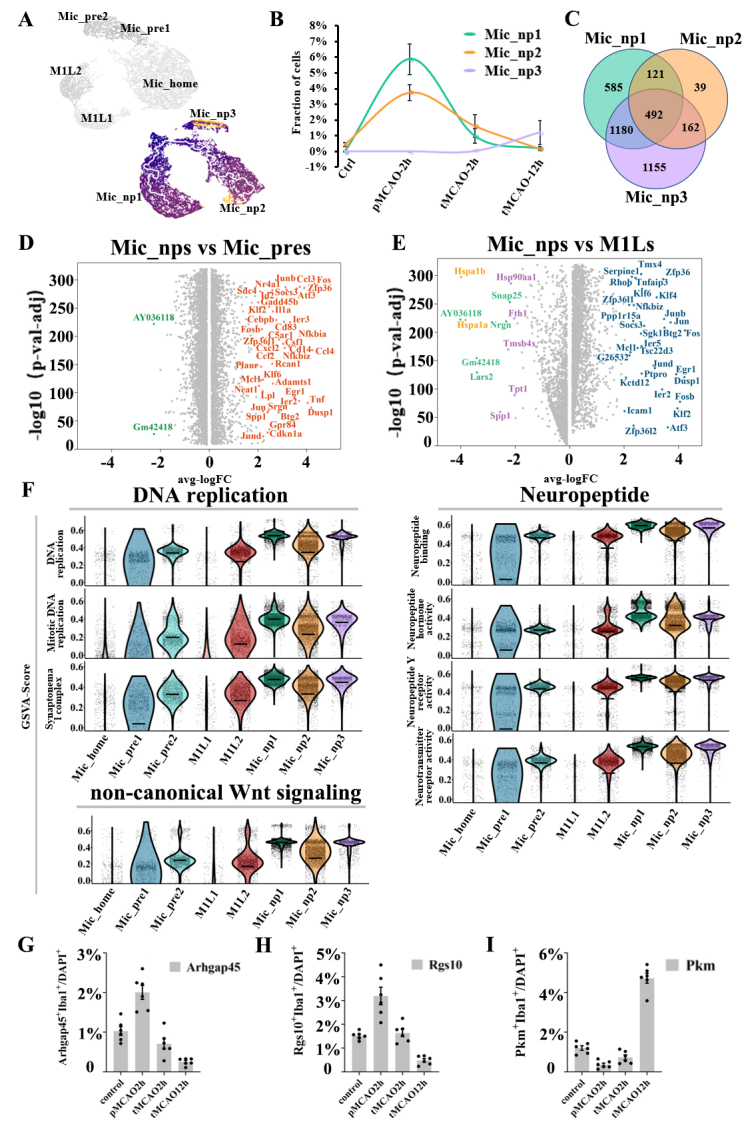
Subpopulation analysis of neuropeptide-associated microglia clusters. (A) Pseudotime plot (from monocle3) shows the differentiation trajectory in Mic_np1, Mic_np2, and Mic_np3 cells respectively. (B) Line plot of the fraction of cells in the Mic_np1, Mic_np2, and Mic_np3 subpopulations over time. (C) Venn plot of the mutual and unique marker genes for the Mic_np1, Mic_np2, and Mic_np3 subpopulations. (D) The volcano plot displays the differentially expressed genes between Mic_np clusters and Mic_pre clusters. The genes are colored according to the subpopulation with the higher expression. (E) The volcano plot displays the differentially expressed genes between Mic_np clusters and Mic_M1L clusters. The genes are colored according to the subpopulation with the higher expression. (F) Violin plot of the functional pathways revealed by Violin plot of the gene set variation analysis (GSVA) in the Mic_np1, Mic_np2, and Mic_np3 subpopulations. (G) Bar plot of the ratio of Arhgap45^+^Iba1^+^ cells (green plus red) compared with DAPI^+^ cells in a 500μm^2^ area surrounding the infarction core (N=6). (H) Bar plot of the ratio of Rgs10^+^Iba1^+^ cells (green plus red) compared with DAPI^+^ cells in a 500μm^2^ area surrounding the infarction core (N=6). (I) Bar plot of the ratio of Pkm^+^Iba1^+^ cells (green plus red) compared with DAPI^+^ cells in a 500μm^2^ area surrounding the infarction core (N=6).

Furthermore, we identified three unique subpopulations that mainly emerged after ischemic stroke. They were quite different from Mic_M1L1 or Mic_M1L2 cells because of the much lower expression level of inflammation genes, and neither subpopulation exhibited M2 polarized markers. These unique cells were characterized by the high activation of neuropeptide Y (Npy/Npyr) and neuropeptide functional pathways (Fig. 2F). As a result, these cells were termed as Mic_np1 (neuropeptide-secretion microglia type1, n= 7992 cells, 21.2%), Mic_np2 (neuropeptide-secretion microglia type2, n= 6720 cells, 17.9%), and Mic_np3 (neuropeptide-secretion microglia type3, n= 1143 cells, 3.8%) (Fig. 6A). The Mic_np1 cluster was characterized by marker genes including Lars2, Arhgap45, and Nrgn expression; and the Mic_np2 cluster was characterized by Rgs10, Ranse4, and Hspa1a expression; and the Mic_np3 cluster was characterized by Pkm, Plp1, and Fth1 expression. The timeline plot showed that these clusters mainly emerged after ischemic stroke, in which the fractions of Mic_np1 and Mic_np2 increased immediately after cerebral ischemia and declined with prolonged reperfusion time, followed by an increase in Mic_np3 cells at 12 hours after reperfusion (Fig. 6B). Compared with the other two subpopulations, Mic_np3 was associated with a lower expression of microglial core genes such as Tmem119 and P2ry12, and the upregulation of Spp1 and Trem2 (Supplementary Fig. 4B). A Venn plot revealed the unique and shared genes among the three clusters (Fig. 6C). For the 10 genes with the highest Moran’s scores, most of the selected genes in Mic_np1 were upregulated along the differentiation trajectory, and those in Mic_np2 were gradually downregulated (Supplementary Fig. 4C). We further compared the differentially expressed genes among Mic_np, Mic_pre, and Mic_M1L clusters (Fig. 6D, Fig. 6E) and the major activated functional pathways according to the results of GSVA enrichment. In these subpopulations, pathways related to DNA replication, neuropeptide activities, and noncanonical Wnt signaling were highly activated (Fig. 6F). The gene of neuropeptide Y was obviously upregulated in Mic_np1 (Supplementary Fig. 4D). Transcription factors analysis showed that Ahctf1 was significantly upregulated in Mic_np clusters, suggesting that it might be an important regulator of Mic_np transformation (Supplementary Fig. 4E). Double immunohistochemical staining further revealed the signature markers of the neuropeptide-associated clusters and the proportional changes which were in line with the sequencing results (Fig. 6G, Fig. 6H, Fig. 6I, Supplementary Fig. 4F, Supplementary Fig. 4G, and Supplementary Fig. 4H).

### Ligand-receptor mediating interactions between microglia and other brain cell populations provide potential neuroprotective targets

**Figure 7.**
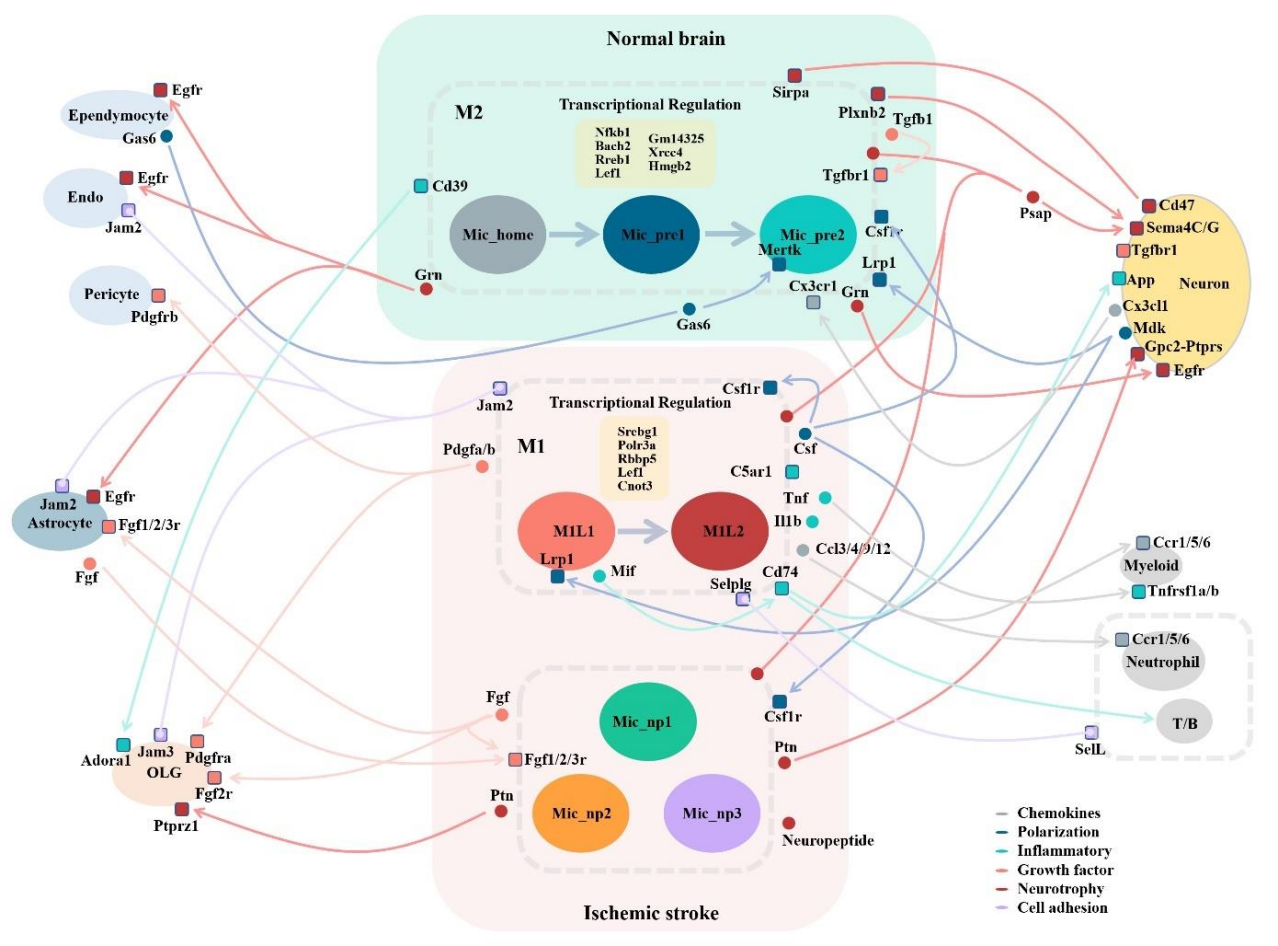
Schematic diagram of the interactions between microglia and other brain cell populations. The small circles represent ligands, and the squares represent receptors. The lines are shown in different colors according to their major functions. The arrows originate from ligands and point to receptors on the cell populations.

Based on the Seurat data containing all the identified brain cell populations, we performed CellphoneDB (v3.0) analysis to detect the stable ligand-receptor interactions during microglial communication and displayed them in a schematic figure (Fig. 7). Among every cell type, before and after ischemia, the interactions with intense association were selected and displayed using dot plots (Supplementary Fig. 5, Supplementary Fig. 6). Under homeostatic conditions, microglia exhibited abundant interactions associated with neurotrophy. Sirpa, Plxnb2, and Grn were secreted to communicate with neurons through Cd47, Sema4C/G, and Egfr receptors respectively. The Grn-Egfr interaction also mediates microglial communication with astrocytes, endo-theliocytes, and ependymocytes in the regulation of lysosomal function, wound healing, and proliferation [26].

The signals from Gas6-Mertk, Csf-Csf1r, and Mdk-Lrp1 interactions might promote the differentiation of homeostatic microglia. After ischemic stroke, there was a significant enhancement in chemokine and inflammatory signaling networks. M1-like microglia secreted Ccl3, Ccl4, Ccl9, Cc12, Tnf, and Il1b to attract myeloid cells, neutrophils, and T/B cells to the CNS system by binding the corresponding chemotactic receptors. Cd74 and its receptors are the major mediators in the inflammatory responses among M1-like microglia, neurons, and T/B cells. The Csf-Csf1r interaction plays a very important role in the differentiation regulation of microglia under both homeostatic and stroke conditions. The Jam2-Jam3 interaction contributed to the cell adhesion of Mic_M1L to astrocytes, oligodendrocytes, and endotheliocytes. Growth factor signaling by the Pdgfa/b-Pdgfra/b interaction were also evident in Mic_M1Ls cells’ communication with oligodendrocytes and pericytes. In contrast, Mic_home cells showed no inflammatory ligand-receptor response with other cell types. Mic_np microglia exhibited higher neurotrophic signals and the secretion of growth factors, as demonstrated by their communication with neurons through Ptn-Ptprs/Gpc2, and Psap-Sema4C/G interactions. They also communicated with oligodendrocytes through Ptn-Ptprs interactions. The Fgf-Fgf1/2/3r interaction was an important mediator for growth factor signaling between Mic_np cells and astrocytes. In summary, microglia exhibited both inflammatory and neurotrophic characteristics in cell communication before and after ischemic stroke. Developing proper methods targeting to these ligand-receptor interactions could be a useful strategy for the regulation of microglial function.

## DISCUSSION

The phenotypic change in microglia after acute ischemic stroke has been debated for decades [27]. In this study, we uncovered the heterogeneity of microglia in brain samples from mice in a homeostatic state and the very early phase after acute ischemic stroke. The scRNA-seq analysis revealed three homeostatic clusters, which were Mic_home, Mic_pre1, and Mic_pre2, and five reactive clusters that mainly emerged after ischemic stroke, naming Mic_M1L1, Mic_M1L2, Mic_np1, Mic_np2, and Mic_np3. This new classification of microglia may facilitate the understanding of microglial phenotype changes in the early phase of acute ischemic stroke.

Our data are not in line with the previous single-cell studies regarding ischemic stroke, because we focus on temporal changes of microglia in a super early time window. The condition of brain cells was distinct. Studies by Guo et al, Zheng et al, and Li et al were based on the brain samples obtained 24 hours after recanalization [15-17]. Moreover, to create the environment of the severe stroke, the duration of artery occlusion in our study was relatively longer (2 hours before recanalization). The TTC, HE, and Nissl tests suggested that the area of unvital tissues progressed fast in the first 12 hours, and the blood reperfusion did not sufficiently stop the deterioration of the ischemic hemisphere after severe ischemic stroke. That the original live cells in the ischemic regions have died and become a part of the infarction core after 24 hours was possible. Additionally, immunofluorescence results showed the evident activation and migration of microglia at 12 hours after blood reperfusion, which further verified the differences between the type of cells captured in our study and those obtained after 24 hours.

Microglia are important regulators for the maintenance of brain homeostasis and neurogenesis in the healthy adult brain. They undertake housekeeping tasks and respond to inflammation irritation [28]. The homeostatic subpopulations in our study showed high expression of microglia core genes such as Tmem119, Cx3cr1, and P2ry12 [29, 30], exhibiting cell proliferation potential, neural trophic support, and low inflammation levels. Numerous signals are released from microglia to regulate the hemostasis of neurons and the blood-brain barrier (BBB), in which Grn-Egfr interaction is very active in the communication. As progranulin having been reported to have neuroprotective effects and the promotion of EGFR-linked signaling pathway following brain injuries also showing beneficial effects, microglia-secreted Grn and their interaction with Egfr might be a potential target [31, 32]. Microglial subsets consisting of cells from sham surgery group were observed in previous studies; here, we further elucidate their heterogeneity [33, 34]. GSVA enrichment suggested that Mic_home was intensely involved in monocyte differentiation pathways. For example, the expression of Tagap, which is important for T(H)17-cell differentiation, was upregulated in Mic_home [35]. In addition, Mic_home cells were less activated in the regulation of neuroinflammation, characterized by a high expression of Hpgd, which could convert resolvins D1, D2, and E1 to their oxo products as a mode of resolvin inactivation [36, 37]. Resolvin D1, D2, and E1 play important roles in the resolution of acute inflammation [38, 39]. Regulated by external differentiation signals such as Csf-Csf1r and Mdk-Lrp1, the level of inflammatory responses increased from Mic_pre1 to Mic_pre2. Mic_pre2 showed highly distinct properties, with most signature genes on the Venn plot, fewer cells in G2/M and S phases, and upregulated genes for protein synthesis and inflammation metabolism. Mic_pre2 was associated with a high-level expression of Wsb1 gene, which is related to pathways including Class I mediated antigen processing and presentation, and the metabolism of proteins. Another marker gene in Mic_pre2 was Ptpro. It has been reported to play critical roles in acute inflammation mediated by T lymphocytes and macrophages [40]. In general, the above heterogeneity of microglia cannot be fully explained by polarization theory, described as changes in the state of microglia after being exposed to an external stimulus. The expression of M2 marker genes was not obvious in Mic_home or Mic_pre cells.

Previous researchers have tended to use M1/M2 phenotypes to describe microglial activation [41]. Classical M1-like polarization, which was used to be seen as harmful polarization state, is characterized by the production of proinflammatory cytokines including IL-1α, IL-1β, IL-6, IL-12, and TNF-α, chemokines, and redox molecules, while alternative M2-like (anti-inflammatory) phenotype is distinguished from M1 by anti-inflammatory transcription profiles such as Il4, Il10, Il13, Arg1, and Ym1 [42]. In recent years, the M1/M2 theory has been challenged because some cells neither obviously expressed the markers for M1 or M2 phenotype, nor behaved biologically homogeneously when they expressed similar polarization markers. The coexpression of M1 and M2 markers in a single cell has also been reported [25]. Although the binary classification of microglia as M1 and M2 is oversimplistic, this theory still be useful as a starting point to understand the complex microglial phenotypes after ischemic stroke. Here, two subpopulations exhibiting M1-polarization tendency manifested by highly expressed inflammatory cytokine or chemokine genes such as Tnf, Lpl, Ccl3, Ccl4, and Csf1 were identified. The following were two main aspects in this study that were different from the classic view of M1-like microglia. First, the M1-like microglia have intrinsic heterogeneity after ischemic stroke, reflected in the different inflammatory states between Mic_M1L1 and Mic_M1L2. In this system, Mic_M1L2 being the earlier dominant M1-like subset, the expression of inflammatory genes such as Tnf, Lpl, and some interleukin genes were considerably high in a portion of M1L2 cells, while the average expression of these genes in all M1L2 cells were lower than those in Mic_M1L1 cells. A possible reason was that microglia captured in the first 2 hours were resident cells that highly reacted to inflammatory amplification, while at 12 hours after blood reperfusion, more distant microglia infiltrated in and exhibited supportive properties; hence, Mic_M1L1 cells were associated with enhanced neural supporting functions, including synapse maturation, presynaptic function, and galactosylceramide biosynthesis, which might have reduced their overall inflammatory reaction. Another possible explanation was that Mic_M1L2 gradually developed binary function and decreased their inflammatory damages. The marker genes in the two subsets were different. For example, Mic_M1L1 was associated with a higher expression of Ccl2, while Mic_M1L2 was with a relatively higher expression of Ccl3 and Ccl4. Second, the M1-like microglia cells should not be regarded as purely harmful cells. Except for the markers of inflammatory factors, both two subsets were associated with protective markers. Rcan1 is one of the significant markers of Mic_M1L2. It had been reported to inhibit NF-kappa B pathway and have a protective role in brain ischemia and reperfusion injury [43-45]. Mydgf was significantly upregulated in Mic_M1L1 cells. Previous studies in the cardiovascular field suggested that Mydgf could protect against pressure overload-induced heart failure, repair the heart after myocardial infarction, inhibits inflammation, and alleviates endothelial injury [46-48]. In addition, the hemostasis and blood coagulation pathways were upregulated in Mic_M1L2, which might be attributed to the activation of the clotting system immediately after blood perfusion. Csf-Csf1r and Mdk-Lrp1 signals were the ligand-receptor couplings intensely involved in the differentiation route between Mic_M1L1 and Mic_M1l2. Lef1 might play an important role in regulating phenotype transformation. This transcription factor was significantly upregulated in the differentiation of both homeostatic and reactive microglia.

Guo et al. suggested that M2-like microglia cells may differentiate more slowly than M1-like microglia [16]. After ischemic stroke, we did not find evident expression of the classic M2-polarization genes (Il4, Il10, Il13, Arg1, and Ym1). Instead, we identified three novel subpopulations without polarization markers in our system. The dot plot showed that most functional genes shared in other clusters, such as Ccl3, Ccr5, Il1a, Egr1, and Grb14 were low-expressed. (Supplementary Fig.2) GSVA analysis implied that the Mic_np clusters were involved in neuropeptide regulation and DNA recovery repair. The three low-inflammation level microglial types emerging in the acute phase manifested high expression of Arhgap45, Rgs10, and Pkm respectively. Arhgap45 is related to histocompatibility antigen (HA-1), MHC, and the innate immune system. The intervention to Mic_np1 through the regulation of MHC class II might be a possible therapeutic target [49, 50]. Mic_np2 was characterized by high expression of Rgs10, Hspa1a, and Hspa1b. Rgs10 can regulate G protein-coupled receptor and inhibit signal transduction, which may resist the injury signaling cascades [51]. Hspa1a and Hspa1b belong to heat shock protein family A (Hsp70). Hsp70 plays an anti-inflammatory role in brain ischemia and protects against cerebral ischemia through its chaperone activity [52-54]. Mic_np3 was associated with high expression of Pkm and Pdgds. Pkm was correlated with energy metabolism, suggesting a high glycolysis level in these cells. The protein encoded by Pdgds catalyzes the conversion of prostaglandin H2 (PGH2) to prostaglandin D2 (PGD2), which functions as a neuromodulator and a trophic factor in the CNS [55, 56]. Furthermore, Mic_np3 is different from Mic_np1 and Mic_np2 in the emerging time and may have some characteristics of disease-associated microglia (DAM), exhibiting a downregulation of microglial core genes (Tmem119 and P2ry12) and upregulation of Spp1 [57, 58]. Previous studies did not report the three subpopulations, which might be attributed to the following reasons. Firstly, our study is the first to explore the very early stage after cerebral ischemia and reperfusion. Secondly, these clusters were temporally specific and might transform into other phenotypes in the long-term pathological process. Thirdly, CD45^+^ was typically used as a marker to filter noninflammatory cells [17]. It was possible that some microglia cells were removed from experimental analysis when they did not show evident expression of Cd45.

From a single-cell perspective, numerous DAMs have been uncovered in brain disorders including multiple sclerosis, Alzheimer’s disease, traumatic brain injury, glioma, and LPS-induced inflammation, while some of the clusters might possess high transcriptional overlap [59]. DAM was characterized by decreased expression of microglial core genes such as P2ry12, Cx3cr1, Csf1r, and Tmem119 and upregulation of neurodegeneration genes including Trem2, Apoe, and Spp1. Among the eight microglial subpopulations, Mic_np3 had similar characteristics [60]. Additionally, new brain resident myeloid cells were identified by scRNA-seq. BAMs are anatomically distinct from microglia and mainly reside in the dura mater, subdural meninges, and choroid plexus. These cells share some same markers with microglia such as Cx3cr1 and Iba1 and are characterized by their high expression of Ms4a7, Lyz2, and H2-Aa [14].

So far, microglia classification during the ischemic stroke has not reached a consensus. In addition to the homeostatic clusters and inflammatory-activated clusters, studies that have investigated microglia subpopulations 24 hours after ischemic stroke identified the neurodegeneration-associated cluster, the IFN pathway activated cluster, the proliferating activated cluster, and the clusters associated with a high chance of survival [14]. A unique “neutrophil-like” subpopulation has also been identified in aged brains after ischemic stroke [14]. In general, the heterogeneity of microglia in different settings suggests that the accurate regulation of microglia should consider the present phenotype of microglia and the proper intervention time. The intervention can be applied to particular transcription factors and ligand-receptor interactions at a specific time point.

Nevertheless, our study has limitations. First, the scRNA-seq analysis was based on mouse brain samples due to the unavailability of brain tissue from ischemic stroke patients. Heterogeneity resulting from differences in the cerebrovascular structure and brain cells of mice and humans was inevitable. Second, because the total brain cortex on the ischemic side was digested for scRNA-seq, we could not clearly distinguish the cells in the penumbra from those in infarcted regions in this model system. Although the percentage of mitochondrial genes in each cell had been restricted during quality filtration, a small number of dysfunctional cells still might be enrolled. Third, long-term changes in phenotypes could not be determined in this study because the time window of our analysis was restricted to the first 12 hours after reperfusion. Fourth, we used mice aged 8 weeks in order to create stable ischemic models. The differences in age, gender, and race of mice can also influence the reaction and heterogeneity of microglia. Finally, the findings in this study need further verification and evaluation by in vitro and in vivo experiments, which will focus on specific subpopulations and their cell markers.

In conclusion, we explored the temporal dynamic heterogeneity of microglia during the early phase of acute ischemic stroke. We identified eight distinct subpopulations displaying neuroinflammation and neural support functions at different stages of the pathological process. Our single-cell dataset could help understand the genetic changes and classification of microglia after ischemic stroke and may facilitate the identification of valuable targets for immunomodulatory treatment at a very early stage of ischemic stroke.

## Supplementary Materials

The Supplementary data can be found online at: www.aginganddisease.org/EN/10.14336/AD.2023.0505.

## Data Availability

The sequencing data supporting the findings of this study are available from the corresponding author upon reasonable request.
