# Supplementary material for: Microglia Exhibit Distinct Heterogeneity Rather than M1/M2 Polarization within the Early Stage of Acute Ischemic Stroke
Source: Aging Dis. 2023 Dec 1;14(6):2284–302. doi: 10.14336/AD.2023.0505 (PMC10676790; doi:10.14336/AD.2023.0505)
Supplement: Supplementary file 1 — The Supplementary data can be found online at: www.aginganddisease.org/EN/10.14336/AD.2023.0505. [file AD-14-6-2284-s.pdf]

SUPPLEMENTARY DATA

Microglia Exhibit Distinct Heterogeneity Rather than M1/M2 Polarization within the Early Stage of Acute Ischemic Stroke

Hongyu Ma[1#], He Li[1,2#], Yongxin Zhang[1#], Yu Zhou[1], Hanchen Liu[1], Hongye, Xu[1], Luojiang Zhu[1], Guanghao Zhang[1], Jing Wang[1], Zifu Li[1], Bo Hong[1], Wang Zhou[1*], Pengfei Yang[1*], Jianmin Liu[1*]

© 2023. Ma H et al. Published online at http://www.aginganddisease.org/EN/10.14336/AD.2023.0505

SUPPLEMENTARY DATA

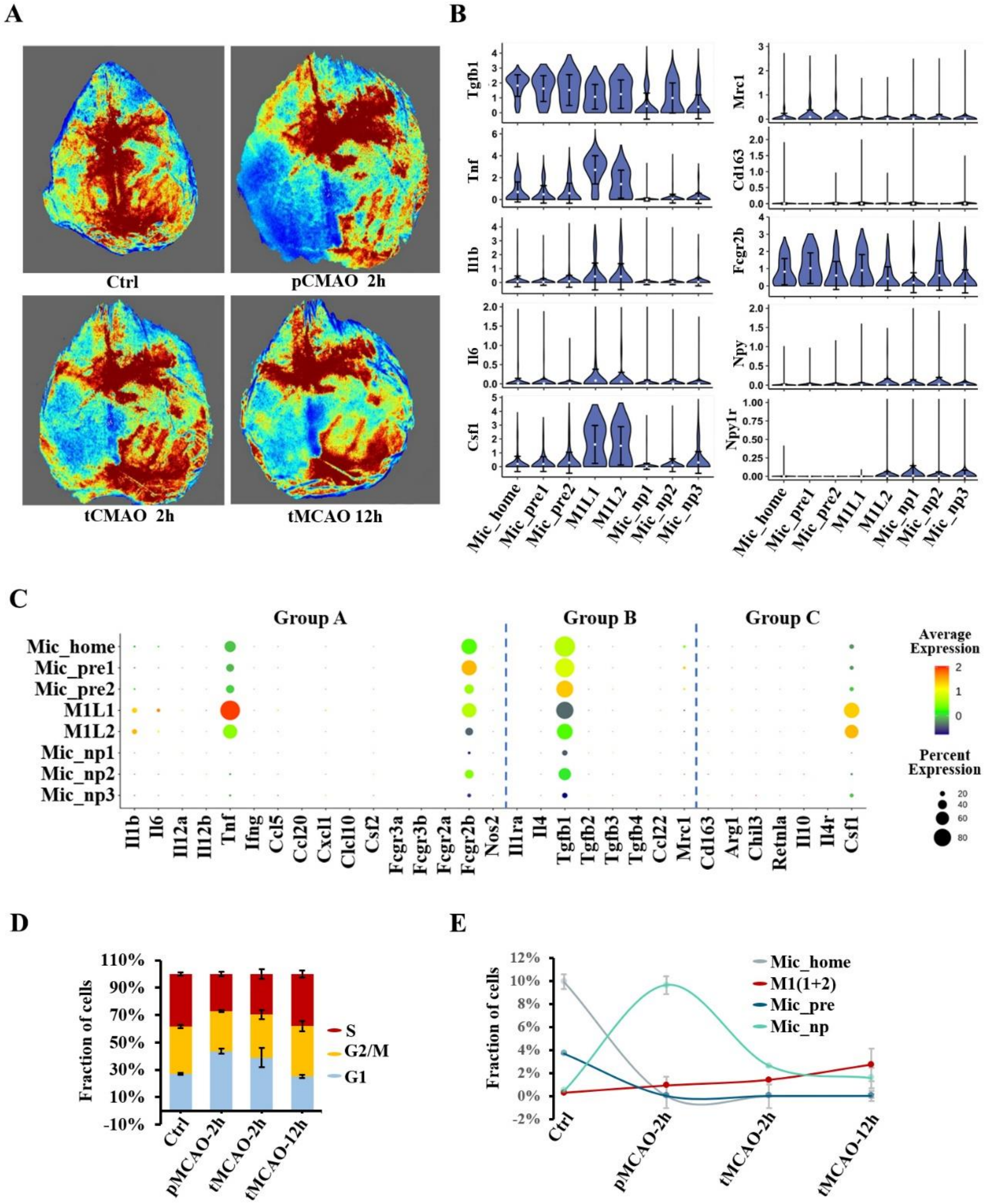

Supplementary Figure 1. Supplementary figures for the identification of subpopulation function. (A) The images of laser speckle flowmetry at the four time points. (B) Violin plot displaying the represented genes related to major microglial function. (C) Dot plot of marker genes for microglial polarization. The genes in groups A, B, and C represent the M1, M2a, and M2b/c phenotypes respectively. (D) The proportion of each cell cycle at four sampling times. (E) Line plot of the temporal change in the fraction of four cell categories.

© 2023. Ma H et al. Published online at http://www.aginganddisease.org/EN/10.14336/AD.2023.0505

SUPPLEMENTARY DATA

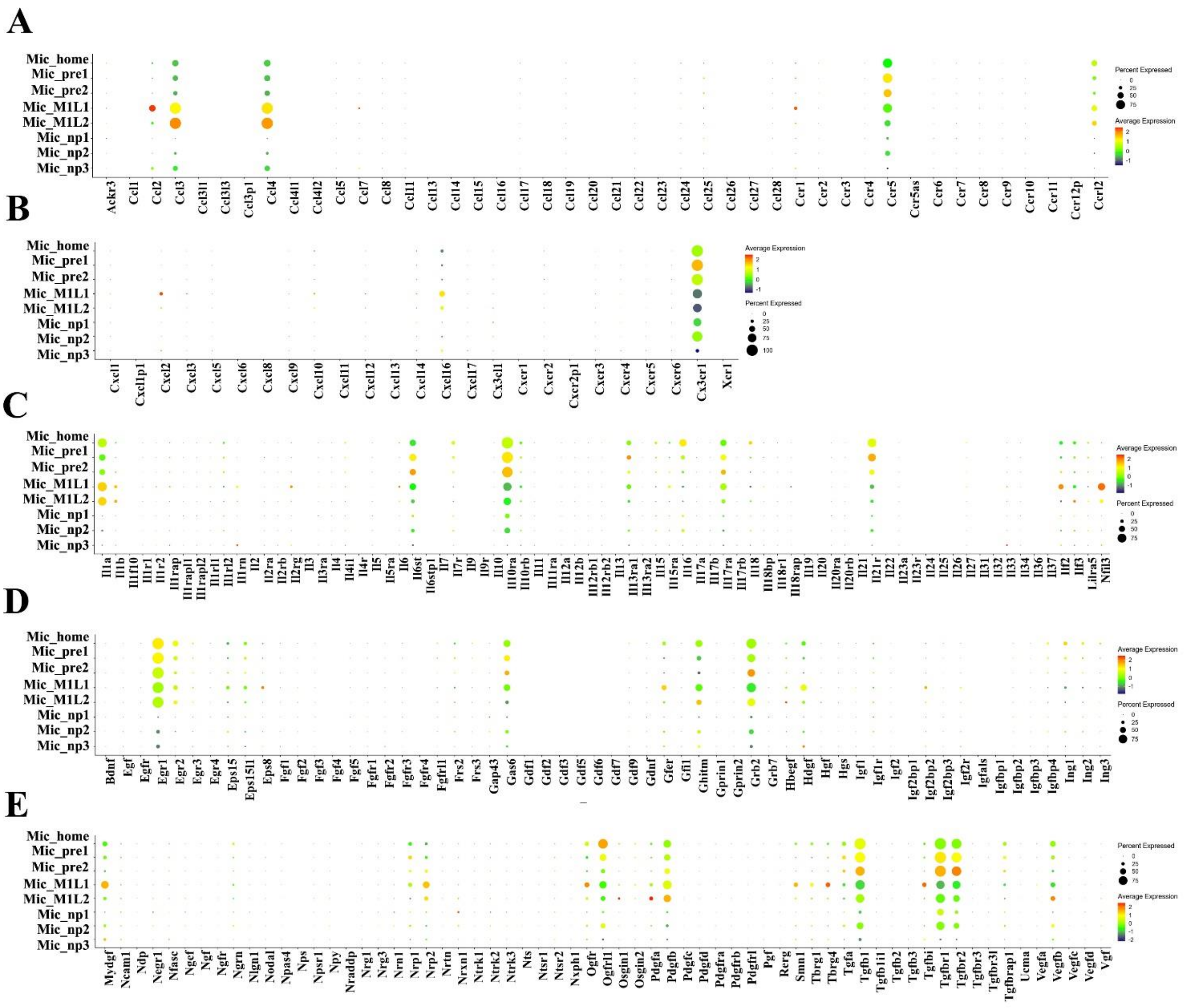

Supplementary Figure 2. Dot plot of major functional genes of microglia. (A) Dot plot displays the expression of genes for CC chemokines. (B) Dot plot displays the expression of genes for CXC, XC, and CX3C chemokines. (C) Dot plot displays the expression of genes for interleukins. (D) Dot plot displays the expression of genes for neural factors, growth factors, and growth-related proteins. (Part1) (E) Dot plot displays the expression of genes for neural factors, growth factors, and neural growth and differentiation. (Part2)

© 2023. Ma H et al. Published online at http://www.aginganddisease.org/EN/10.14336/AD.2023.0505

SUPPLEMENTARY DATA

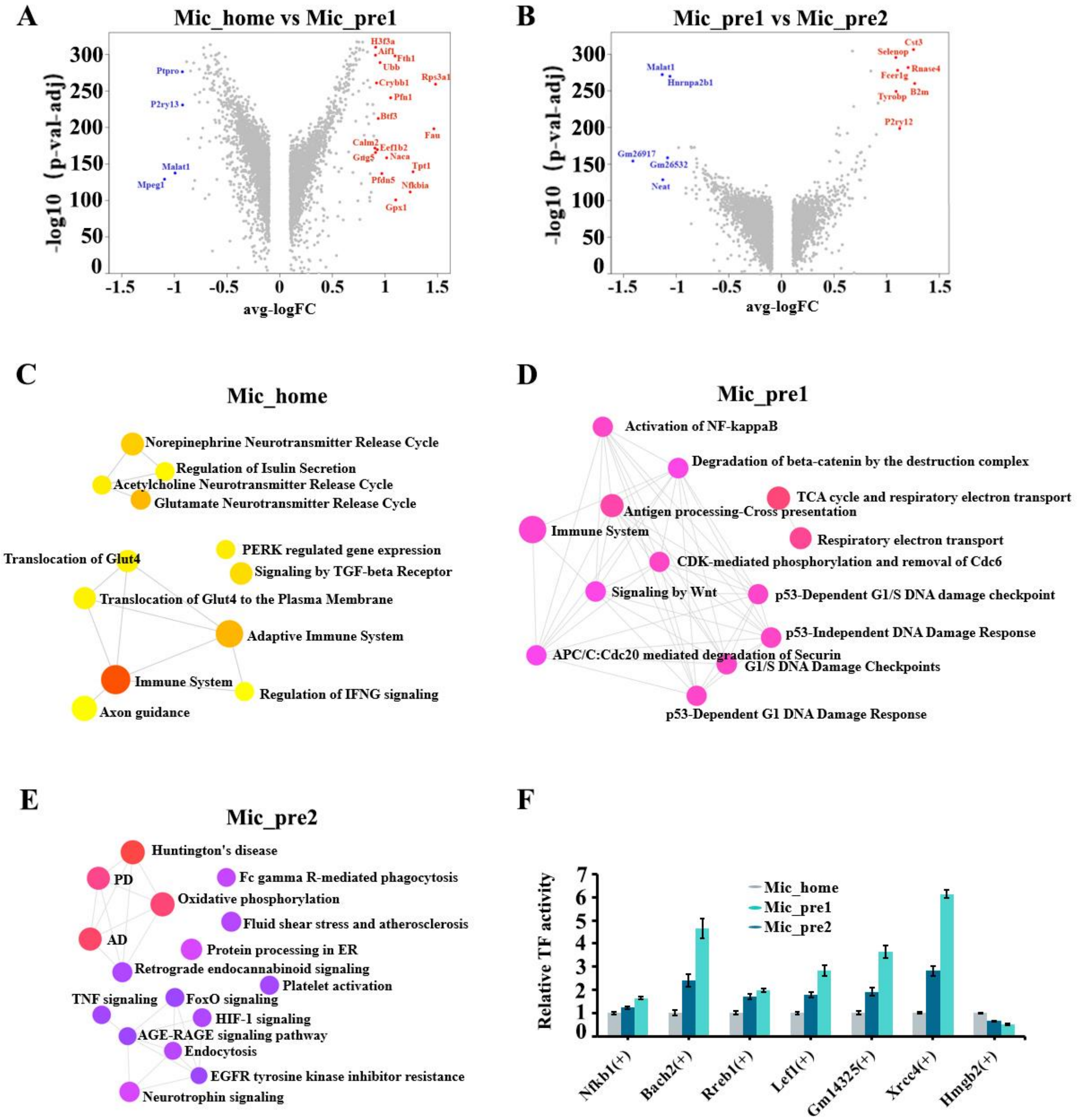

Supplementary Figure 3. Supplementary figures for function comparison among homeostatic microglial subpopulations. (A) Volcano plot to compare the differential genes between Mic_home and Mic_pre1 clusters. Red dots represent upregulated genes in Mic_home compared with Mic_pre1, while blue dots represent the downregulated. (B) Volcano plot to compare the differential genes between Mic_pre1 and Mic_pre2 clusters. Red dots represent upregulated genes in Mic_home compared with Mic_pre1, while blue dots represent the downregulated. (C-E) Ontology network of the major activated pathways and their interaction in Mic_home, Mic_pre1, and Mic_pre2 subpopulations respectively. (F) Relative activity of the highly expressed transcription factors (TF) in Mic_pre1 and Mic_pre2 cells compared with Mic_home cells. The values of the bars denote the ratio of Mic_pre1 and Mic_pre2 cells compared with Mic_home cells.

© 2023. Ma H et al. Published online at http://www.aginganddisease.org/EN/10.14336/AD.2023.0505

SUPPLEMENTARY DATA

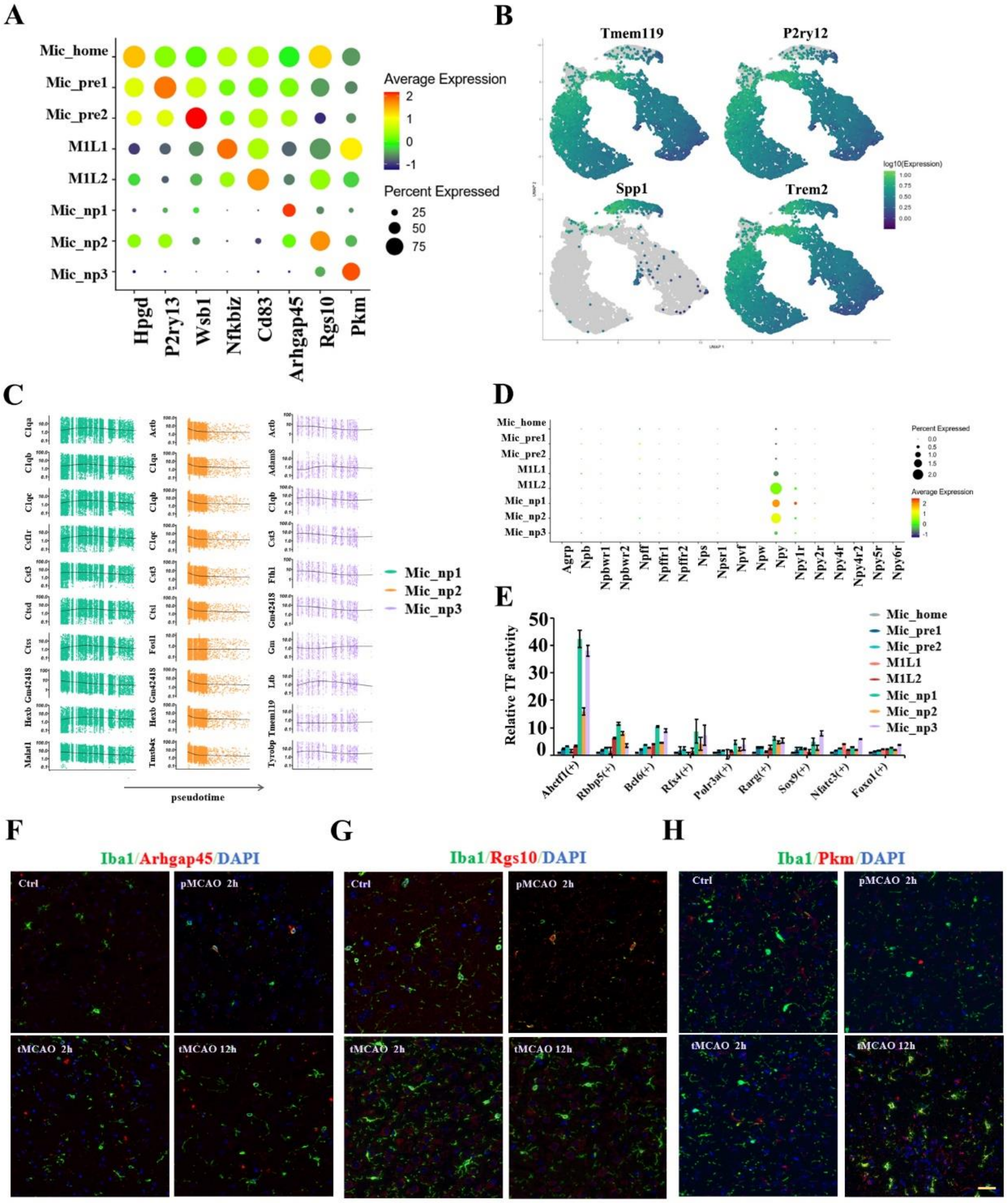

Supplementary Figure 4. Supplementary figures for the characteristics of neuropeptide-associated microglial subpopulations. (A) Dot plot of the markers for each subpopulation used for double-immunofluorescent staining. (B) The expression of core microglial marker genes and disease-associated microglia genes in the Mic_np3 subpopulation. (C) Expression of the ten genes with the highest Moran's I score alongside the pseudotime trajectory in the Mic_np1, Mic_np2, and Mic_np3 subpopulations. (D) Dot plot of the neuropeptide genes. (E) Relative activity of the high expressed transcription factors in Mic_np2 and Mic_np3 cells compared with Mic_np1 cells. (F) Double immunofluorescence staining of Mic_np1 cells at each sampling time. Coronal brain sections are all stained with anti-Iba1(green, representing microglia) and DAPI (blue, representing cell nuclei) antibodies. Mic_np1 are marked by Arhgap45 (red). (N=3) The yellow bar represents 100μm. (G) Double immunofluorescence staining of Mic_np2 cells at each sampling time. Coronal brain sections are all stained with anti-Iba1(green, representing microglia) and DAPI (blue, representing cell nuclei) antibodies. Mic_np2 are marked by Rgs10 (red). (N=3) The yellow bar represents 100μm. (H) Double immunofluorescence staining of Mic_np3 cells at each sampling time. Coronal brain sections are all stained with anti-Iba1(green, representing microglia) and DAPI (blue, representing cell nuclei) antibodies. Mic_np3 are marked by Pkm (red). (N=3) The yellow bar represents 100μm.

© 2023. Ma H et al. Published online at http://www.aginganddisease.org/EN/10.14336/AD.2023.0505

SUPPLEMENTARY DATA

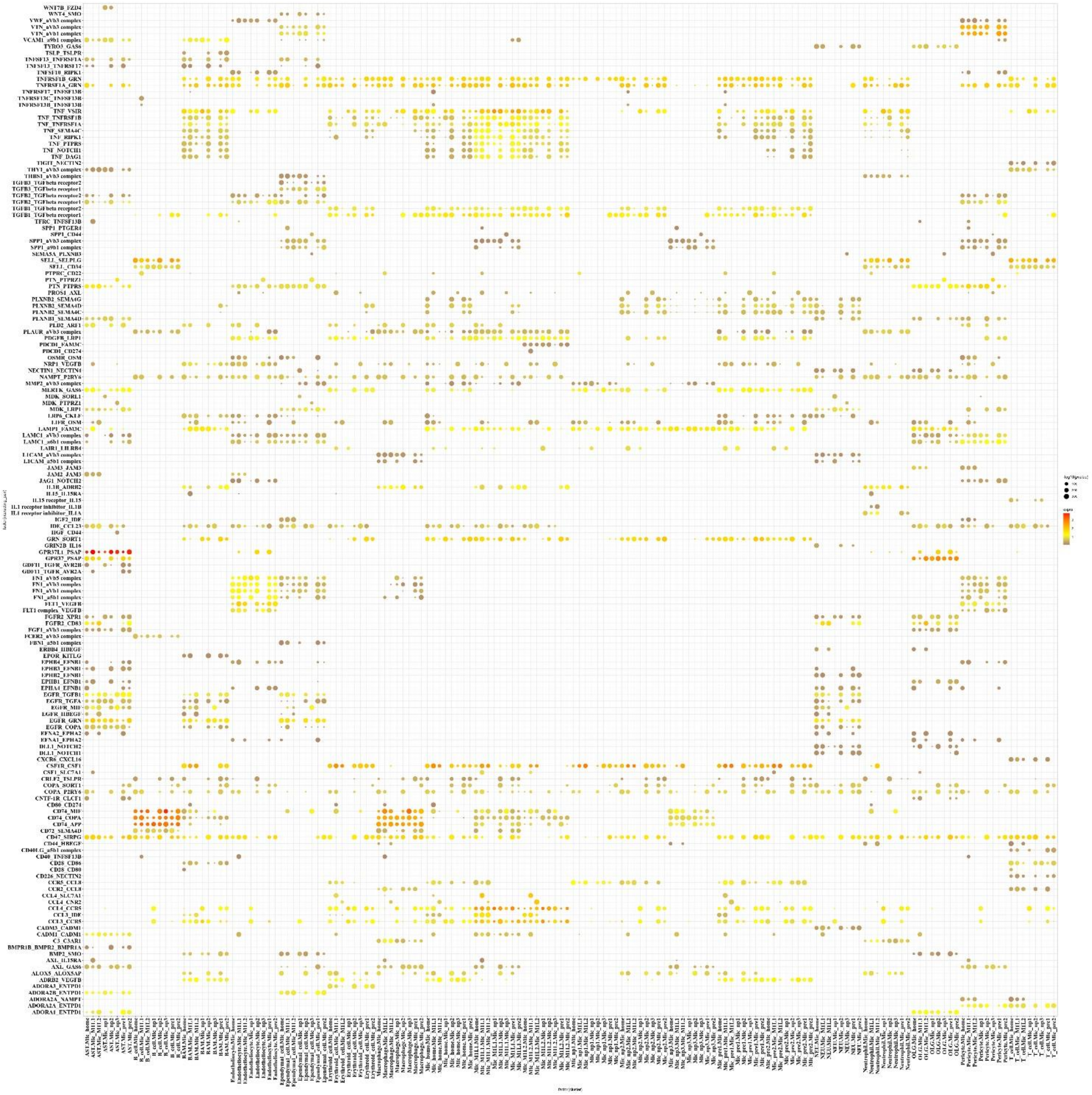

Supplementary Figure 5. Dot plot of the major activated ligand-receptor couplings between the subpopulations of microglia and other brain cell populations under control state.

© 2023. Ma H et al. Published online at http://www.aginganddisease.org/EN/10.14336/AD.2023.0505

SUPPLEMENTARY DATA

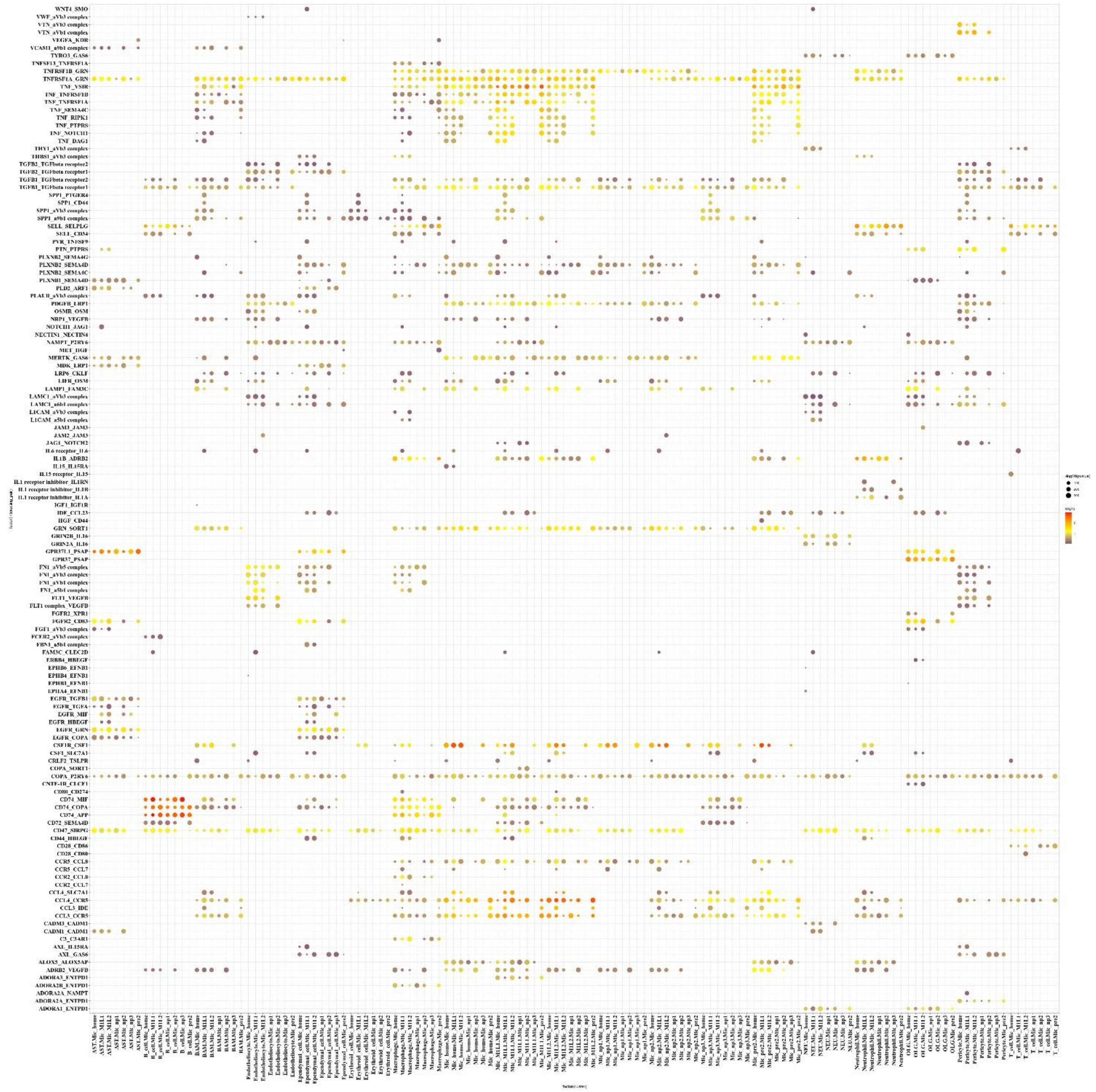

Supplementary Figure 6. Dot plot of the major activated ligand-receptor couplings between the subpopulations of microglia and other brain cell populations after ischemic stroke.

© 2023. Ma H et al. Published online at http://www.aginganddisease.org/EN/10.14336/AD.2023.0505
